# Nutritional, Clinical and Sociodemographic Profiles of Spanish Patients with Amyotrophic Lateral Sclerosis

**DOI:** 10.3390/nu16030350

**Published:** 2024-01-25

**Authors:** Sandra Carrera-Juliá, José M. Estrela, Mario Zacarés, Mari Ángeles Navarro, María Jesús Vega-Bello, José Enrique de la Rubia Ortí, Mari Luz Moreno, Eraci Drehmer

**Affiliations:** 1 Department of Nutrition and Dietetics, Catholic University of Valencia San Vicente Mártir, 46001 Valencia, Spain; sandra.carrera@ucv.es; 2 Department of Physiology, University of Valencia, 46010 Valencia, Spain; jose.m.estrela@uv.es; 3 Department of Basic and Transversal Sciences, Catholic University of Valencia San Vicente Mártir, 46001 Valencia, Spain; mario.zacares@ucv.es (M.Z.); angeles.navarro@ucv.es (M.Á.N.); 4 Department of Anatomy and Physiology, Catholic University of Valencia San Vicente Mártir, 46001 Valencia, Spain; mj.vega@ucv.es; 5 Department of Nursery, Catholic University of Valencia San Vicente Mártir, 46001 Valencia, Spain; joseenrique.delarubi@ucv.es; 6 Department of Health and Functional Assessment, Catholic University of Valencia San Vicente Mártir, 46001 Valencia, Spain; eraci.drehmer@ucv.es

**Keywords:** amyotrophic lateral sclerosis, clinical characteristics, demography, nutritional intake, Spanish population

## Abstract

Amyotrophic lateral sclerosis (ALS) is a chronic and progressive neurodegenerative disease that leads to the loss of motor neurons. The dietary intake of ALS patients is thought to influence the prognosis and progression of the disease. The aim of this study was to examine the nutritional, clinical and sociodemographic characteristics of ALS patients in Spain. A cross-sectional descriptive study with demographics, clinical anamnesis and anthropometric assessment was carried out. Nutritional intake was recorded and compared with dietary reference intakes (DRI). Forty subjects (25 males; 15 females) aged 54.7 ± 10.17 were included in the study. The mean weight and height were 67.99 ± 8.85 kg and 167.83 ± 8.79 cm, respectively. Clinical phenotype, time to diagnosis, year of onset and family history were not associated with the place of origin. Clinical phenotype had no influence on time of diagnosis. Caloric and protein intakes were adequate, while carbohydrate, vitamin B8 and iodine intakes were significantly lower than the DRI. Lipids; vitamins B1, B2, B3, B5, B6, B12, C and E; sodium; phosphorus; and selenium intakes were significantly higher than the recommended nutritional standards. ALS patients, who are homogeneously distributed throughout our national territory, should modify their dietary habits to minimize ultra-processed products and prioritize foods rich in healthy fats and fiber.

## 1. Introduction

ALS is a neurodegenerative disease characterized by the impairment of both upper motor neurons in the cerebral cortex and lower motor neurons in the brain stem and spinal cord [1]. The hallmark symptomatology includes muscle atrophy and weakness, loss of neuromotor control, decreased ability to perform voluntary movements, and difficulties in oral communication, swallowing and breathing [2]. Initially regarded as a pure motor disorder, ALS is now believed to be a multisystemic disease.

The highest incidence of ALS is typically observed between the ages of 50 and 75 [3]. However, there are cases of the disease occurring in individuals under the age of 25, which is classified as juvenile ALS [4]. The incidence of the disease is slightly higher in males than in females [5].

ALS can be categorized into three clinical phenotypes. Familial ALS accounts for approximately 5–10% of cases [6]. Spinal ALS (65–70%) is more prevalent in males and affects the upper and/or lower limbs [7]. Bulbar ALS (20%) is more frequently observed in females and produces weakness in the bulbar, oropharyngeal and orofacial muscles [8].

The worldwide prevalence and incidence rate of ALS in the general population is estimated to be 4.42 (95% CI: 3.92–4.96) per 1,000,000 people, with an incidence rate of 1.59 (95% CI: 1.39–1.81) per 1,000,000 person-year [9]. In Spain, there are few sociodemographic studies on ALS. In 2019, the prevalence in Spain was eight cases per 100,000 inhabitants, equivalent to 3717 patients, according to reports from the Luzón Foundation ALS Observatory (www.ffluzon.org, accessed on 5 February 2020).

There is currently no cure for ALS, and the only two approved pharmacological treatments are Riluzole (Rilutek^®,^) and Edaravone (Radicava^®^) (Mitsubishi Tanabe Pharma Corporation (MTPC), Osaka, Japan) [10]. The mean survival rate for ALS patients typically falls within the range of two to five years since the onset of symptomatology [11]. Only 25% of patients survive beyond five years after diagnosis, although 5–10% may survive more than 10 years [12]. Maintaining a healthy nutritional diet is considered a crucial factor in enhancing patients’ long-term survival and influencing the progression of the disease [13]. Patients may experience dysphagia, making it difficult to consume solid and/or liquid foods, which can increase the risk of nutritional deficiencies and weight loss [14]. In addition, decreased appetite and altered salivary secretions also contribute to inadequate caloric and nutritional intake. Malnutrition, which negatively impacts prognosis, may promote disease progression, and reduces survival rates [15]. ALS pathophysiology causes disturbances in redox homeostasis, neurodegeneration, and neuroinflammation [16]. Therefore, nutrients with antioxidant and anti-inflammatory properties are of particular interest. Additionally, an analysis of dietary fiber intake is recommended as patients with ALS present alterations in their intestinal transit. Despite this, there are a limited number of studies that have examined the nutritional intake of patients affected by ALS, and even fewer comparing their nutritional intake with standard nutritional recommendations [17].

Based on these considerations, the objective of our study was to describe the clinical and sociodemographic characteristics of Spanish patients affected by ALS, their nutritional intake, and the nutritional adequacy and deficiencies or excesses compared to the DRI (dietary reference intake).

## 2. Materials and Methods

### 2.1. Type of Study and Participants

A descriptive cross-sectional study was conducted. The Spanish foundation for the promotion of ALS (FUNDELA) helped to recruit patients meeting the following inclusion criteria: age over 18, diagnosed with ALS according to the “El Escorial” criteria with a minimum of six months of disease duration, and able to consume food orally [18]. Exclusion criteria included patients who were pregnant or breastfeeding, had a history of alcohol or drug consumption, had undergone gastrectomy, relied on total or partial enteral nutrition through percutaneous endoscopic gastrostomy (PEG), or had a diagnosis of dementia.

### 2.2. Ethical Aspects

This study and all the procedures related to the participants were approved by the Human Research Ethics Committee (CEIH) of the University of Valencia (ref. number H1479983999044). The study was registered at www.clinicaltrials.gov (accessed on 18 May 2018) (number NCT03489200). The entire study followed the guidelines established by the declaration of Helsinki. All participants signed an informed consent form containing details on the procedures and nature of the study.

### 2.3. Procedures

All procedures were carried out at the clinical facilities of the Catholic University of Valencia San Vicente Mártir (Valencia, Spain). The collaboration of the family and caregivers was involved in those cases where the patient had mobility or communication difficulties.

#### 2.3.1. Clinical and Sociodemographic History

A personal interview with each patient was conducted to gather sociodemographic information and assess their clinical background. This included details such as place of residence, age, clinical phenotype, symptomatology onset, date of diagnosis, personal medical history, family history and other relevant factors.

To calculate the mean duration between symptomatology onset and the time of ALS diagnosis, we relied on data obtained from the patients’ clinical records and the information gathered during the clinical anamnesis.

#### 2.3.2. Anthropometric Valuation

Anthropometric assessments in the study adhered to the international society for the advancement of kinanthropometry (ISAK) protocols. All measurements were conducted using validated and calibrated equipment. Measurements were taken by a certified ISAK level III anthropometrist.

A SECA (Hamburg, Germany) portable clinical scale was used to measure body weight in patients with normal mobility. This scale has a range of 150–200 kg and a precision of 100 g, ensuring accurate weight measurements. In cases where participants had limited mobility, an electronic SECA 954 chair-type scale was used with a maximum capacity of 300 kg and a precision of 100 g.

To accurately measure height to a precision of 0.1 cm, a SECA 220 model stadiometer was employed. In situations where participants were unable to stand upright for height measurement, the height data in their clinical records was used.

Body mass index (BMI) was calculated and categorized as normal weight (≥18.5–24.99 kg/m^2^); underweight (≤18.5 kg/m^2^); grade I overweight (25–26.9 kg/m^2^); grade II overweight (27–29.9 kg/m^2^); or obesity (≥30 kg/m^2^).

#### 2.3.3. Analysis of Nutritional Intake

A four-day dietary diary was maintained to record the intake of solid and liquid foods. It was required that one of the four days documented fell on a Saturday or Sunday. The decision to collect dietary information over a span of four days was based on the belief that it would provide a more comprehensive understanding of the patient’s typical nutritional intake and would reduce potential bias associated with selecting just a single day for data collection [19]. In these diaries, patients recorded the number of meals consumed, amount of food consumed, ingredients used and preparation method for each dish. The patients were given information on weight, portions and household measurements standardized by the Spanish society of community nutrition and healthy eating guide (SENC), thus facilitating maximum accuracy in terms of the weight and volume of food consumed. With this information, an analysis of patients’ nutritional intake using “Nutrición y Salud^®^” software version 2.0 (University of Granada, Granada, Spain) was carried out.

We assessed whether the intake was nutritionally adequate according to available recommendations. In the absence of specific recommendations for patients with ALS, the results of the percentage of macronutrient distribution were compared to the nutritional objectives for the Spanish population established in the SENC consensus [20]. The micronutrient results were compared with the dietary reference intake (DRI) for the Spanish population, established in 2010 by the “Federación Española de Sociedades de Nutrición, Alimentación y Dietética”. Individual energy requirements were calculated using the Harris–Benedict equation [21] and the physical activity coefficient recommended by the WHO.

#### 2.3.4. ALSFRS-R Test

The revised ALS functional rating scale (ALSFRS-R) test was performed in all participants. It is a sensitive, accurate and reproducible scale which assesses functional ability considering the domains of impairment: bulbar, upper limb, lower limb and respiratory [22].

#### 2.3.5. Statistical Analysis

Statistical analysis was carried out using R software (2020 version). The results are presented as mean ± standard deviation, number of patients or percentage compared to the total sample number. The normality of the variables was assessed using the Shapiro–Wilk test and assumption of equal variances was checked using Bartlett’s test. The Kruskal–Wallis non-parametric test was used for variables that did not meet the criteria of normality and homoscedasticity. All effects were considered significant when *p* was ≤0.05.

## 3. Results

### 3.1. Clinical and Sociodemographic Profile

The sample included 40 patients: 25 males and 15 females (62.5% and 37.5%, respectively). The mean age was 54.7 ± 10.17 years, with an age range of 37–80 (Table 1).

The patients had a mean weight of 67.99 ± 8.85 kg and a mean height of 167.83 ± 8.79 cm. Specifically, the average weight in men was equal to 72.02 ± 7.46 kg, and in women, it was equal to 61.28 ± 6.75 kg. The mean BMI was 24.14 ± 2.56 kg/m^2^. In men, the mean BMI was equal to 24.55 ± 2.28 kg/m^2^, and in women, it was equal to 23.47 ± 2.94 kg/m^2^.

The minimum time elapsed until the moment of diagnosis was 0 years, which indicated that the diagnosis was made in the same year the symptoms appeared. The maximum time elapsed until diagnosis was four years, which indicated that some patients obtained a diagnosis of ALS four years after the onset of the first symptoms. The time elapsed from the onset of symptoms to the time of this study ranged from one to eight years.

The score obtained for the ALSFRS-R was 40.13 ± 0.70.

**Figure 1:**
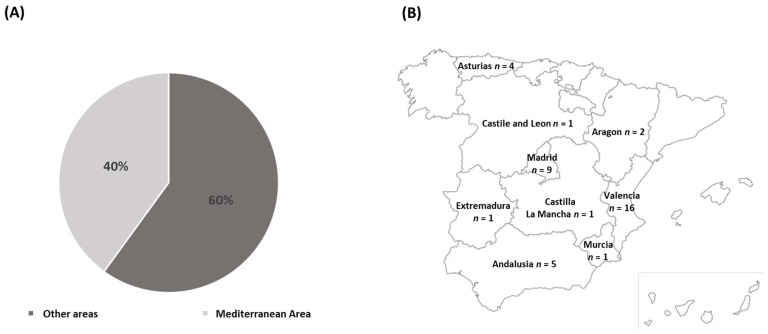
Geographical distribution of the patients. (**A**) Distribution of the patients between the Mediterranean area and others. (**B**) Distribution of the patients according to the Spanish Communities.

Out of the total sample, 16 (40%) were from the Mediterranean Zone and 24 (60%) were from other areas of Spain: Andalusia, Aragon, Estremadura and Castile Leon (Figure 1A). Specifically, the origin of the patients was as follows: 4 from Asturias, 1 from Castile and Leon, 2 from Aragon, 9 from Madrid, 1 from Extremadura, 1 from Castilla-La Mancha, 5 from Andalusia, 1 from Murcia and 16 from Valencia (Figure 1B).

**Figure 2:**
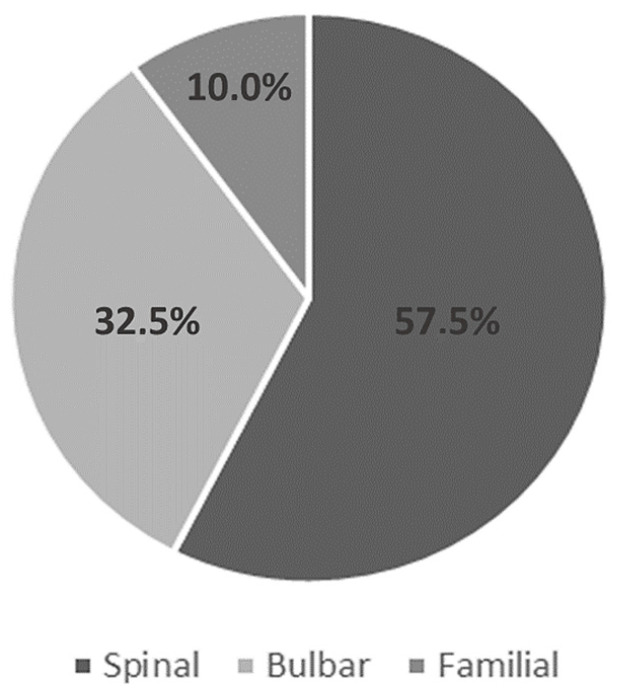
Classification of the study sample according to the clinical ALS phenotype.

The predominant clinical phenotype was spinal (23 patients, 57.5%); 13 patients (32.5%) presented the bulbar phenotype and 4 (10%) the familial phenotype (Figure 2).

No association was found between place of origin and the following variables: clinical phenotype (*p* = 0.378), diagnosis time (*p* = 0.193), year of onset (*p* = 0.234) and family history (*p* = 0.503). There was also no association between clinical phenotype and time of diagnosis (*p* = 0.225).

### 3.2. Analysis of Nutritional Intake

Table 2 shows our analysis of the usual mean nutritional intake of the Spanish ALS patients who participated in the study. Each nutrient is accompanied by its DRI to determine whether the identified mean intake was nutritionally adequate or not.

#### 3.2.1. Energy and Macronutrients

The mean caloric intake was 2211.67 kcal, which falls within the range recommended by the DRI (1875–3000 kcal).

The mean distribution of macronutrients showed that the intake of carbohydrates (40.18%) was significantly lower than the minimum recommended intake (50% of the caloric content). The intake of proteins was 18.58%, thus complying with recommendations (10–20%).

The mean intake of total lipids was 41.23%, which was significantly higher than the recommended maximum of 35%. Additionally, the intake of saturated fatty acids (13.31%) was significantly higher than the recommended range of 7–8%. Similarly, the intake of polyunsaturated fatty acids (11.96%) was significantly higher than the 5% recommended. The mean intake of monounsaturated fatty acids was 15.92%, which was significantly lower than the 20% recommended.

The mean intake of cholesterol was 351.14 mg, which was slightly higher than its DRI (<300 mg), but not significantly higher than what is recommended. The mean fiber intake of 21.31 g did not meet the minimum value recommended (25 g).

#### 3.2.2. Micronutrients

The mean intake of vitamin B1 (1.48 mg) was slightly higher than the maximum recommended value of 1.2 mg. The intakes of riboflavin (2.16 mg) and niacin (31.75 mg) were significantly higher than the DRI (1.6 and 18 mg, respectively). Similarly, the intake of vitamin B5 was 5.67 mg, which was significantly higher than its DRI (5 mg). Our findings were similar in the case of pyridoxine, with a mean intake of 2.40 mg, which was significantly higher than the maximum recommended value of 1.6 mg. In contrast, the mean nutritional intake of vitamin B8 of 8.06 µg was significantly lower than the DRI of 30 µg.

There was a slightly deficient intake of vitamin B9 (275.89 µg) compared to its DRI (300 µg). The intake of vitamin B12 (8.03 µg) was higher than its DRI (2 µg).

The intakes of vitamin C (94.60 mg) and vitamin E (17.68 mg) were significantly higher than their DRI (70 mg and 15 mg, respectively). The mean intake of vitamin A was 837.53 µg, slightly higher than its DRI of 600–700 µg.

The mean intake of vitamin D was 5.11 µg, which met the DRI, with a value close to the minimum recommended of 5 µg.

The mean intake of calcium was 941.57 mg, which met its DRI (900–1000 mg). However, the intake of phosphorus (1384.26 mg) was much higher than its DRI (700 mg).

Sodium presented an intake of 2255.10 mg, much higher than its DRI (1300 mg). The intake of magnesium was equal to 330.71 mg and met its DRI. Iron had an adequate dietary intake of 16.55 mg. Zinc presented a mean intake of 10.40 mg, meeting its DRI. The intake of iodine (84.49 µg) was significantly below its DRI (150 µg). Selenium presented a mean intake (78.51 µg) that was significantly higher than its DRI (55 µg).

All indicated values of micronutrients and their DRIs refer to the average age of the patients in this study.

## 4. Discussion

### 4.1. Clinical and Sociodemographic Profile

In this study, a higher incidence rate of ALS was observed in males, consistent and significant based on the literature [23], which suggests that estrogens may play a (still undefined) protective role in women [24]. Our findings regarding the mean age align with previous studies noting that ALS incidence rates increase with advancing age. Age is considered a risk factor for ALS, as is the presence of the disease in the family [6]. It is worth noting that in this study, there was a case of a younger patient deviating from the typical age range.

The predominant clinical ALS phenotype observed in the Spanish population was spinal. No significant association was found between patients’ place of origin and their clinical phenotype, indicating that geographical location within the country did not impact the incidence rates or clinical presentation of the disease. Similarly, no significant relationship was observed between patients’ origin and their clinical phenotype regarding the time of diagnosis. This suggests that the Spanish health system works effectively. In addition, the sociodemographic data showed a relatively even distribution of ALS across different regions of Spain.

### 4.2. Nutritional Intake Profile

#### 4.2.1. Energy and Macronutrients

The observed compliance with DRI guidelines regarding caloric intake could be attributed to the body weight of the sample population. The importance of maintaining an appropriate caloric intake is underscored by its potential benefits in preserving BMI and nutritional status, and decreasing the probability of malnutrition, thereby positively impacting the overall survival rates [25]. However, while the overall quantity of the diet was deemed satisfactory, a qualitative assessment revealed that the consumption of carbohydrates and lipids did not align with the recommended DRI. Consequently, it could be inferred that the regular dietary patterns of these patients lacked proper macronutrient distribution, indicating an imbalance.

Specifically, the considerable deficit in carbohydrate intake below the recommended minimum is noteworthy. This is particularly significant because neural tissue needs a consistent and sufficient supply of glucose to function optimally [26]. A reduced contribution of carbohydrates could potentially impair brain performance and negatively affect the functionality of the nervous system [27]. In fact, in ALS, it is also known that glucose transport and metabolism can be progressively impaired in the motor neurons [28]. Thus, a modified ketogenic diet (e.g., enriched in mid-chain fatty acids as an alternative source of energy) could also complement a combined therapy [29].

However, it is important to note that the positive effects associated with fat consumption are contingent upon maintaining a balanced distribution of fatty acids in the lipid profile, which was not observed in this study [30]. The excessive intake of saturated fatty acids has been linked to increased inflammation [31], while an excess of polyunsaturated fatty acids can impact the lipid profile of brain cell membranes and potentially elevate lipid peroxidation [32]. On the contrary, other research indicates that higher intakes of polyunsaturated fats are associated with a reduced risk of ALS compared to lower intakes, which may be attributed to the potential reduction in excitotoxicity triggered by glutamate [33]. The association between monounsaturated fatty acids and ALS appears to be of lesser significance [34].

An adequate intake of proteins is positive, as previous research has linked excessive protein consumption to an elevated risk of ALS [35]. Neurodegeneration and the pathophysiology of this disease contribute to additional muscle mass loss [36]; thus, sufficient protein intake would aid in preserving muscle quality.

The mean fiber intake in this study fell below the minimum recommended level. Consequently, we recommend increasing fiber consumption, as patients with ALS are more susceptible to constipation [37]. Certain studies have found that patients with ALS face a higher risk of intestinal dysbiosis compared to the general population [38]. A study conducted on Korean ALS patients found a correlation between fiber intake, disease progression rate and survival time. These results suggest that higher fiber intake was negatively correlated with pro-inflammatory cytokines, suggesting that the intake of fiber might contribute to slow disease progression [39]. It should be noted that while in this study, the average carbohydrate intake was below the RDA, and simple carbohydrates were not determined, the deficient intake of fiber is an aspect to consider. Fiber is a nutrient that contributes to reducing the glycemic response following the consumption of carbohydrate-rich foods. This is due to its ability to slow gastric emptying and/or glucose absorption in the intestinal mucosa, thereby attenuating the peak blood glucose response and insulin demand. This is relevant for ensuring proper glucose metabolism [40].

#### 4.2.2. Micronutrients

Because thiamine and pyridoxine play crucial roles in the synthesis of neurotransmitters, maintaining an adequate intake of these vitamins to support optimal neurotransmitter synthesis and function is essential [41]. The intake of biotin was significantly lower than the DRI. Vitamin B8 serves as a cofactor for carboxylase enzymes involved in the production of ATP, which is necessary for neurons, oligodendrocytes and myelin synthesis [42].

Given that dietary factors have the capacity to influence oxidative stress (OS), an analysis was conducted on the nutritional intake of vitamins known for their antioxidant potential: B2, B3, C, A and E. In all cases, their intake was higher than the DRI, which could confer resistance against OS, a mechanism involved in the pathophysiology leading to motor neuron death [43]. However, it is also important to note that one specific study found a correlation between high levels of vitamins A and E and an elevated risk of ALS (in this study, the pro-oxidant effects of natural antioxidants, if given in excess, are known) [44].

Additionally, the finding of a slightly deficient intake of vitamin B9 holds significance, as it has been observed that folic acid levels can become deficient during the later stages of the disease [45]. This vitamin plays a role in the process of myelination, and, along with B12, it is also involved in maintenance of the cell membrane and the production of acetylcholine. In a SOD1^G93A^ mouse model, the ingestion of B9 was found to protect motor neurons against neuroinflammation and apoptosis [46]. As the disease progresses, it would be advisable to include foods rich in vitamin B9, such as leafy green vegetables.

This study revealed an intake of vitamin B12 higher than the DRI. This finding could be attributed to the fact that none of the subjects followed a vegan diet, which typically limits the consumption of this vitamin due to its primary presence in animal-based foods [47].

An adequate contribution of vitamin D is essential in neurodegenerative diseases [48] as it protects motor neurons, enhances axon regeneration and reduces microglia-induced neuroinflammation [49]. Vitamin D’s role in bone metabolism is well known and must be considered in ALS patients, who exhibit a greater deterioration of bone health compared to the general population [50].

Although the mean intake of calcium was deemed appropriate, this study identified a significantly higher intake of phosphorus than the DRI, which could be considered potentially detrimental due to its contribution to an imbalance between these two essential nutrients. These two minerals play an essential role in phospho-calcium metabolism, neuromuscular function and mineralization [51]. This excessive intake of phosphorus could be attributed to a high consumption of meat derivatives or soft drinks, which often contain phosphoric acid as a preservative [52]. This study found that certain patients consumed soft drinks and meat derivatives on a daily basis, not complying with the recommendations. Consequently, ALS patients could benefit from a diet including calcium-rich foods such as dairy products, cruciferous vegetables and nuts, and limiting ultra-processed products rich in phosphorus and its variants.

A high intake of sodium can be considered harmful as its excess can contribute to an increased risk of arterial hypertension [53], a clinical comorbidity that increases cardiovascular risk and complicates prognosis [54]. Consequently, moderating the use of salt when cooking and limiting the consumption of ultra-processed products that contain high amounts of “hidden salt” is recommended.

Adhering to the recommended intake of magnesium would be beneficial due to the role of this mineral as an enzymatic cofactor in metabolic reactions within the nervous system and muscle. Similar to magnesium, maintaining adequate iron levels is crucial. Iron deficiency can increase the risk of developing iron deficiency anemia, while excessive iron levels can elevate the risk of neurodegenerative diseases [55] by promoting OS and neuroinflammation [56]. Similarly, an adequate intake of zinc is also crucial because it promotes neurogenesis and neurotransmission. Zinc is associated with OS and neuroinflammation due to its role as an antioxidant, anti-inflammatory, and immunomodulatory agent [57]. Indeed, a low zinc intake has been related to an increased risk of neurodegenerative diseases [58].

This study found that patients had a significantly lower intake of iodine than that recommend by the DRI. Studies conducted on mice found that iodine deficiency is associated with restricted myelin synthesis and delayed neuronal maturation [59].

Finally, selenium is known for its antioxidant properties. Nevertheless, in this study, selenium intake was higher than the recommended levels, and it has been reported that excessive selenium intake may lead to the increased production of free radicals and thus cause motor neuron damage [60].

### 4.3. Limitations of the Study

The limited sample size of this study is insufficient to reach definitive conclusions, but, in light of the observations, further research is warranted. Moreover, instructing patients to complete multiple dietary diaries in order to gather information on nutritional intake over an extended period would be of interest. Furthermore, it is important to note that there are currently no specific DRIs for patients affected by ALS, who have specific nutritional needs.

Due to the lack of similar studies conducted in Spain, a comparison was made between the results of this study and findings from other geographical regions characterized by distinct dietary patterns and gastronomic cultures. Eating habits differ within the same country, and this should be considered, since our patients came from different areas of the country.

The dietary-nutritional software used in this study did not provide data on the consumption of simple carbohydrates. It also does not allow for the determination of the average intake of antioxidant nutrients, such as polyphenols and anti-inflammatory nutrients such as omega-3 fatty acids (eicosapentaenoic acid (EPA) and docosahexaenoic acid (DHA)). These nutrients should be considered in future studies because ALS is associated with oxidative stress and inflammation, and they are characteristic components of the Mediterranean Diet. Furthermore, exposure to sun as a source of vitamin D was not considered. Biochemical analyses of serological values for vitamins and minerals would provide valuable insights into the impact of nutritional intake on ALS patients.

## 5. Conclusions

Spinal ALS is the predominant clinical phenotype in Spain, and the geographical distribution of the disease shows a relatively homogeneous pattern. The place of origin of Spanish ALS patients, presence of family history, time of diagnosis and year of onset of the disease do not influence the clinical phenotype. In addition, clinical phenotype does not affect the time of diagnosis. Patients were recommended to amend their dietary habits to align with the DRIs by reducing their intake of saturated fat, phosphorus and sodium and increasing their consumption of heart-healthy fats, complex carbohydrates, high-quality proteins and fiber. These changes could help to improve the symptomatology and slow down the progression of the disease. Our findings could also help patients in other countries and cultures with different dietary habits. To achieve the goal of improving the dietary intake of patients with ALS, we believe that nutrition education is an interesting methodology for teaching the science of nutrition to patients and their families. This should be carried out by specialized healthcare professionals such as dietitians and nutritionists. In this way, patients can make positive changes in their dietary habits and develop greater adherence to a healthy eating pattern in the long term. They could also learn fundamental concepts about food composition, healthy culinary techniques, foods rich in antioxidants and anti-inflammatory properties, among others. If necessary, the prescription of nutritional supplementation could be useful, especially when the intake of a particular nutrient is deficient and cannot be ensured through meals.

## Figures and Tables

**Table 1:** General and clinical characteristics of the study sample.

Variable	Total (*n* = 40)
Sex	
Male	25
Female	15
Age (years)	54.7 ± 10.17
Age (years),	
Minimum–maximum	37–80
Weight (kg)	67.99 ± 8.85
Men	72.02 ± 7.46
Women	61.28 ± 6.75
Height (cm)	167.83 ± 8.79
BMI (kg/m^2^)	24.14 ± 2.56
Men	24.55 ± 2.28
Women	23.47 ± 2.94
Time of diagnosis (years),	
Minimum–maximum	0–4
Duration of ALS (years),	
Minimum–maximum	1–8
ALSFRS-R	40.13 ± 0.70

ALSFRS-R: revised ALS functional rating scale; SD: standard deviation; BMI: body mass index. The descriptive analysis values are shown as sample number, mean ± SD and minimum–maximum interval. Time to diagnosis (years) refers to the time elapsed from the onset of symptoms until diagnosis, with the minimum time being 0 years (year of diagnosis and onset of symptomatology were coincident) and the maximum time being four years (diagnosis four years from onset of symptomatology). The duration of ALS (years) refers to the duration of the disease since the onset of symptoms until the time the study began, presenting the minimum and maximum time elapsed.

**Table 2:** Description of the mean nutritional intake of the study sample.

Nutritional Variable	Mean	SD	DRI
Energy (kcal/day)	2211.67	305.86	1875–3000
Carbohydrates (%/day)	40.18 **	4.20	50–55
Proteins (%/day)	18.58	2.43	10–20
Lipids (%/day)	41.23 **	3.91	30–35
Monounsaturated fatty acids (%/day)	15.92 **	0.12	20
Polyunsaturated fatty acids (%/day)	11.96 **	0.21	5
Saturated fatty acids (%/day)	13.31 **	0.20	7–8
Cholesterol (mg/day)	351.14	214.11	<300
Fiber (g/day)	21.31	6.33	25–35
B1-Thiamine (mg/day)	1.48 *	0.61	1–1.2
B2-Riboflavin (mg/day)	2.16 **	1.06	1.2–1.6
B3-Niacin (mg/day)	31.75 **	7.82	14–18
B5-Pantothenic acid (mg/day)	5.67 *	1.31	5
B6-Pyridoxine (mg/day)	2.40 **	1.66	1.2–1.6
B8-Biotin (µg/day)	8.06 **	5.04	30
B9-Folic acid (µg/day)	275.89	91.67	300
B12-Cyanocobalamin (µg/day)	8.03 **	5.36	2
C-Ascorbic acid (mg/day)	94.60 **	50.00	60–70
A (µg/day)	837.53	637.53	600–700
D (µg/day)	5.11	3.17	5–10
E (mg/day)	17.68 **	3.88	15
Sodium (mg/day)	2255.10 **	528.80	1200–1500
Potassium (mg/day)	2998.29	779.89	3100
Calcium (mg/day)	941.57	283.98	900–1000
Phosphorus (mg/day)	1384.26 **	290.11	700
Magnesium (mg/day)	330.71	109.26	300–350
Iron (mg/day)	16.55	6.29	9–18
Zinc (mg/day)	10.40	5.06	7–10
Iodine (µg/day)	84.49 **	29.55	150
Selenium (µg/day)	78.51 **	25.98	55

SD: standard deviation; DRI: dietary reference intake. * *p* < 0.05, ** *p* < 0.01 vs. DRI.

## Data Availability

All data generated or analyzed during this study are included in this published article.
